# Tagging Efficiency of ^99m^ Tc-SC Radiolabeled Alternative Gastric Emptying Meals: A Quantitative Study

**DOI:** 10.1055/s-0042-1757255

**Published:** 2022-09-09

**Authors:** Deepak Kumar Pal, Dhananjay Kumar Singh, Satyawati Deswal, Anurag Pathak

**Affiliations:** 1 Department of Nuclear Medicine, Dr. Ram Manohar Lohia Institute of Medical Sciences, Lucknow, Uttar Pradesh, India

**Keywords:** gastric emptying scan, gastroparesis, sulfur colloid

## Abstract

**Objective** The aim of this study was to know the tagging efficiencies of technetium-99m labeled sulfur colloid (99mTc-SC) with different meals.

**Materials and Methods** Egg white sandwiches are the gold standard for gastric-emptying scan (GES); thus, an egg white omelet labeled with ^99m^ Tc-SC is taken as a standard meal. For evaluation, we included four meals, bread and butter, instant oatmeal, idli, and chapatti, and all meals were prepared by labeling them with ^99m^ Tc-SC. After preparation, food articles were chopped with the help of a metal fork and mixed in simulated gastric fluid. Four samples were taken simultaneously from each food article and analyzed for 1 to 4hours after agitation within the centrifuge. The samples were filtered and separated from the sediments and supernatants. We analyzed the activity in each sample before and after filtration.

**Results** The mean values of labeling efficiency in per cent of various meals were obtained. There was no significant difference in labeling stability for egg whites, chapatti, and idli meals labeled with ^99m^ Tc-SC from 1 to 4hours as their *p* -value (p>0.05) was insignificant.

**Conclusion** Radiolabeled chapatti and idli with ^99m^ Tc-SC show higher labeling stability, while oatmeal and bread and butter samples show relatively low stability. Thus, for GES, chapatti and idli labeled with ^99m^ Tc-SC can be used as alternatives to eggs for vegetarian people or for those allergic to eggs.

## Introduction

A gastric emptying scan (GES) is a scintigraphy test that is done in patients having gastroparesis, a syndrome characterized by delayed GE with symptoms such as vomiting, belching, bloating, distensions, fullness, and early satiety. In the case of early emptying, also known as dumping syndrome, GES are preferred by gastroenterologists. This GES is done with the help of radiolabeled test meal, which is currently used an egg white sandwich (as gold standard), but the problem arises in vegetarian patients, who are allergic to eggs or uncomfortable in ingesting eggs; thus, in these patients alternative meals are required and in our study, we are trying to find out the suitable alternative of egg meal so that we can use them in the vegetarian patient for GES. 1

Earlier, the chicken liver is used for the GES. However, the procedure of labeling chicken liver with technetium-99m labeled sulfur colloid ( ^99m^ Tc-SC) is too burdensome and could not be possible in the current working environment in any nuclear medicine department. Thus, alternatives of nonvegetarian meals were tried by many researchers, but the task is not yet fulfilled up to the optimum levels. Various pieces of literature show the use of a variety of food articles for GES, such as milk, peanut butter sandwiches, burritos, and muffins. Despite the assortments of various meals, variations in the cooking method for labeling with ^99m^ Tc-SC were also observed, such as adding ^99m^ Tc-SC before and after cooking in various meals. 2

To overcome the variations, lack of consistency, and standardization, a panel of expert gastroenterologists from the American Neurogastroentrology and Motility Society and nuclear medicine physicians from the Society of Nuclear Medicine and Molecular Imaging (SNMMI) has published a consensus guideline for solid meal GES in the year 2008. 3

The consensus recommendation standardized the following parameters for the procedure: the frequency of imaging, test meal used, duration of the procedure, and normative control values of GE. 3 The regulation of technique is required to allow the referring physician to interpret the results of GES from different centers confidently. But the presence of single standardized meal results in reduced utilization of this diagnostic procedure in current situations.

The reliable results of GES are dependent upon how efficiently the test meal is labeled with the ^99m^ Tc-SC. Unstable labeling will result in the false and poor interpretation of GES. Thus, we have the requirement of finding a radiolabeled test meal with equal or optimum labeling stability of that of liquid egg whites.

Thus, in this study, we include four different test meals other than liquid eggs white omelet, which is taken as standard as recommended by SNMMI for GES; these are chapatti, idli, bread and butter, and oatmeal. The choice of different meals was dependent upon the ease of preparation of the meal and easy availability of them.

## Materials and Methods

This prospective study compared the assessed alternative meals with the standard egg meal used for GES for gastroparesis and other gastric-related pathologies. This in vitro study was performed in the nuclear medicine department at DRRMLIMS, Lucknow, from August 2019 to September 2021.

### The Inclusion Criteria

1. The alternative meals to be used in the study are selected on the basis of ease of preparation.
2. While choosing the material, it was decided based on the easy availability of the food article in the market.

The following materials are required for the experiment purpose:

1. Dose calibrator (CAPINTEC, INC. CRC-ULTRA).
2. Remi-303 centrifuge machine.
3. Centrifuge vials.
4. Whatman Filter paper 12.5cm diameter.
5. Plastic funnels.
6. Plastic syringes 2,5,10mL.
7. Gloves 7.5 No.
8. pH paper narrow range.
9. Beakers.
10. Hydrochloric acid.
11. Pepsin enzyme
12. Sulfur colloid kits.
13. Eluted ^99m^ Tc
14. 0.9% of normal saline.
15. Distilled water.
16. Food articles (eggs, bread and butter, oats, whole wheat flour, idli mix packet).
17. Refined vegetable oil.
18. Utensils (pan, bowel, idli stand, pressure cooker, tava, spoon, fork, etc.).

### Labeling Method

^99m^ Tc-SC 925 MBq (25mCi) was added to different meals for preparing sample meals. Sample meals included were chapatti, idli meal, bread and butter, and oatmeal. Cooked egg whites labeled with ^99m^ Tc-SC served as control. The required radioactivity was mixed in liquid egg whites taken and blends well for uniform distribution. A pan was taken, and after that, one teaspoon of oil was sprinkled in a pan and heated the pan. After 1minute, the blended mixture was poured into the pan and waited until it gained the solid-state, cooked on both sides, and removed on a plate and the test meal was ready to use. Packed instant oatmeal and idli batter were prepared by adding water containing the required radioactivity. Packed instant oatmeal was prepared by cooking them in a pan on flame for 3minutes with continuous string. Idli meal was prepared by brushing the mold with refined vegetable oil for easy lodging of them after cooking and then the batter was poured into the mold with the help of a spoon, the mold was placed into the steamer after 15 to 20minutes and then it was removed from the steamer and allowed to cool for 5minutes and removed from the mold with the help of spoon and the idli meal was ready for use. Chapatti of wheat flour was prepared by adding the radioactivity at the time of preparation of dough and further the dough was rolled, and chapatti was prepared by baking them on flame. Bread and butter sample was prepared by adding the required radioactivity into the butter at melting. When the butter regained the semisolid state, it spread upon the bread, and a sandwich of bread and butter was prepared. The cooking of every meal was ensured before using them further in the experiment.

### Stability Test Method

Each meal is allowed to cool at room temperature after its preparation. The food articles were chopped into small pieces with the help of a metal fork to simulate chewing and for gastric stimulation, the food articles were kept under simulated gastric fluid prepared by 1N hydrochloric acid (HCL) solution and pepsin enzyme solution.

At the same time, four samples each of 2g were taken from two food articles, assessed their activity inside the dose calibrator (CAPINTEC, INC. CRC-ULTRA), and placed inside the centrifuge vials in the acidic medium and homogenized with the simulated gastric fluid. The contents of the centrifuge vial were 3mL of water, 2mL of 1N HCl, and 2mL of pepsin enzyme solution to the test tube. Similarly, four samples were agitated for the period of 1 to 4hours and subsequently centrifuge at 2,000rpm for 2minutes before the assessment of readings inside the dose calibrator at 1-hour interval, and first test tube of both samples was removed from the centrifuge machine filtered them with the help of Whatman filter paper using Y type funnel in test tube stand. We separated the supernatant and sediments and assessed their activity before and after filtration; we washed the sediments with 2mL of saline during filtration. Similarly, the experiment was repeated until we covered all the samples and repeated the testing method for four consecutive experiments.

Calculated labeling efficiency (LE) of different samples as:



## Results

After finding the results for the percentage of LE of various meals concerning time for experiment first, second, third, and fourth are given below in the tables. As we compare the results, we can see that percentage binding of eggs white; chapatti and idli meals are good concerning other two taken alternatives ( Table 1 ).

**Table 1:** Percentage of solid binding at different time points

Sl. no.	Meals labeled with ^99m^ Tc sulfur colloid	Labeling stability % at 1 hour	Labeling stability % at 2 hours	Labeling stability % at 3 hours	Labeling stability % at 4 hours
1.	Egg white omelet	97.62±2.18	96.45±3.85	93.67±3.40	91.56±3.80
2.	Chapatti	92.01±1.51	89.39±3.79	86.73±3.91	84.47±5.28
3.	Idli meal	89.44±5.26	86.79±6.10	82.63±7.38	77.75±9.11
4.	Instant oatmeal	71.13±8.38	66.97±11.00	63.65±11.53	58.46±11.03
5.	Bread and butter	71.70±7.12	68.66±6.28	65.87±6.24	61.62±5.03

**Fig. 1:**
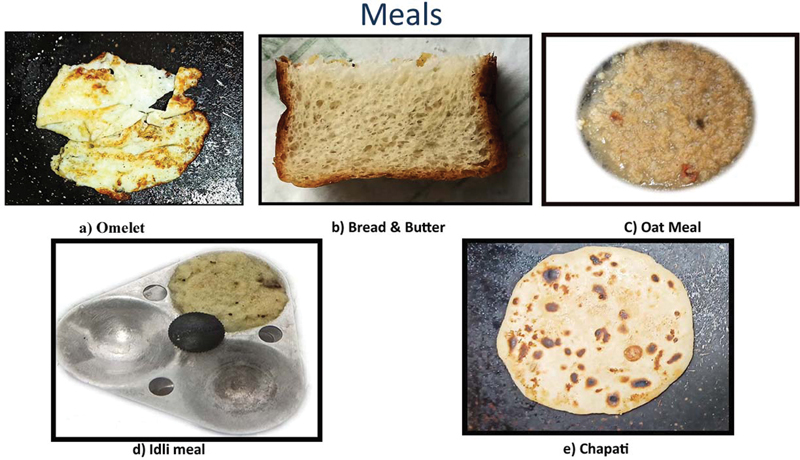
Percentage of solid binding at different time points.

The results after the assessment of LE are plotted in Fig. 1 . The standard taken is egg white omelet, radiolabeled chapatti gives excellent result, and idli meal has also been used as an alternative vegan meal as its result is also up to the optimum level. The percentage of activity bound to the solid phase of eggs white, chapatti, and idli was 97.62±2.18, 92.01±1.51, and 89.44±5.26, respectively, at 1 hour; 96.45±3.85, 89.39±3.79, 86.79±6.10, respectively, at 2hours; 93.67±3.40, 86.73±3.91, and 82.63±7.38, respectively, at 3hours; and 91.56±3.80, 84.47±5.28, and 77.75±9.11, respectively, at 4hours. The other alternatives, oatmeal and bread and butter, were not given the desired results, so both could not suggest the percentage bound in the solid phase of oatmeal and bread and butter was 71.13±8.38, 71.70±7.12, respectively, at 1 hour; 66.97±11.00, 68.66±6.28 at 2hours, 63.65±11.53, 65.87±6.24 at 3hours; and 58.46±11.03, 61.62±5.03, respectively, at 4hours. We found the percentage activity bound in solid food to be less than 80%, but the variability between these two samples was significant between 1 and 4hours.

## Discussion

GES is mainly performed in nuclear medicine to detect gastroparesis or any type of gastrointestinal disorder. GES is accomplished by radiolabeling the solid or liquid component of a meal and then measuring the radioactivity in the stomach with time. It has been considered the gold standard test for measuring the rate of GE due to its noninvasive, physiologic, and quantifiable properties. 5

After getting the results by applying the statistical tests (ANOVA and Tukey multiple comparison tests) on the experimental data, we learned that there is no significant difference between the egg white and chapatti as well as between chapatti and idli meal as in both cases ( *p* >0.05) and all three meals gave us good LE percentage suggesting that chapatti and idli meal could be an excellent alternative to egg white meal. We were further looking at the data, and we observed that there is no significant difference between bread butter meal and oatmeal ( *p* >0.05). However, both showed relatively poor LE compared with other meals.

Various factors are involved in the efficient binding of the radiotracer, which in our case was ^99m^ Tc-SC. Those factors are in the following text.

### Uniformity of Radiotracer Inside Meal

It is essential that the molecules of radiolabel should be uniformly distributed and remains associated with the particles of solid during exposure to gastric fluid. 2 In our results, the radiotracer molecules could get the spaces with uniform distribution in the egg meal, chapatti, and idli because of the excellent ^99m^ Tc-SC mixing during meal preparation. Even the prior study of idli meal done by Somasundaram et al 6 indicates that uniform distribution of radiotracer in meal gives us better binding and efficiency results. Their study of radiolabeling stability showed that the mean percentage activity remaining bound to the idli meal at 1, 2, 3, and 4hours of treatment with SGF was 98.2, 98.2, 97.8, and 96.8%, respectively, thus providing a strong binding of the ^99m^ Tc-SC with food particles. Moreover, only a part of the meal was radiolabeled in the egg white sandwich meal and the chicken liver meal (egg white in egg white sandwich and chicken liver in the latter). In contrast, the rest of the meal was unlabeled (bread slices and jam in egg white sandwich as well as broth in chicken liver meal). 7 8 This means the radiotracer molecules were not uniformly distributed. In our experiment also bread and butter is a case in which the bread slices are not radiolabeled, and the butter has the radiotracer in it, and it has shown poor radiolabeling efficiency in comparison to other meals, which reads out to be 78.68, 76.66, 74.28, and 68.89% at the time interval of 1, 2, 3, and 4hours, respectively (mean values of all four experiment). Similar is the case with oatmeal in which hot water had radiotracer molecules bound to it and oats were not having a uniform distribution of ^99m^ Tc-SC within it, leading to relatively poor results of radiolabeling efficiency, which are found to be 78.75, 75.54, 70.22, and 67.81% at the time interval of 1, 2, 3, and 4hours, respectively.

### State of Meal

The state of the meal also plays a specific role in determining radiolabeling efficiency of a particular meal. If it is a solid meal, there are much more chances to get better efficiency. We get relatively lower efficiency in the case of liquid meals because atoms in solid are closely packed, or we can say are compact and make efficient bonds within them, contrary to liquids where the higher movement of atoms leads to the relatively weak binding. Even few studies have stated that solid GES are more delicate for detecting gastroparesis than liquid GE, which is the rare requirement of GE liquid studies. 9 In our results, oatmeal, the only semi-solid meal taken, showed comparatively poor radiolabeling efficiency than other solid meals. However, in some studies, liquid nutrient meals have been suggested as an alternative to standard egg white based on the results found on gastric retention. 10 11

### Method of Preparation

In a study done by Knight et al, 12 it was concluded that the stability of a radiolabeled solid test meal must be performed for GES according to its constituents and the preparation method. The addition of ^99m^ Tc-SC to a selected meal should be chosen correctly, which means whether radiotracer is to be added before, during, or after cooking. An experiment showed that radiolabeling by adding ^99m^ Tc-SC to whole eggs before microwave cooking resulted in a significantly higher LE than radiolabeling when the ^99m^ Tc-SC was squirted on eggs after microwave cooking. 4 Here, we can also say that when radiolabeling was done before cooking, radiotracer molecules got uniformly distributed, resulting in higher radiolabeling efficiency. When ^99m^ Tc-SC was spilled over the cooked meal, it could not spread uniformly. Earlier, we discussed the uniform distribution of radiolabeled particles in a meal on the radiolabeling efficiency. It can be interpreted that it is the method of preparation that has a connection with the distribution of radiotracer molecules in a meal. Whenever radiotracer is added to a meal before its cooking, there would be the chances of uniformity in which radiotracer would get in meal, and the radionuclide ^99m^ Tc-SC after cooking would result in nonuniformity in the meal. Our results also show that egg white, chapatti, and idli meal have shown us better results. The radiolabeling efficiency percentage for egg white was found to be 99.07, 98.85, 95.34, and 90.04 at 1, 2, 3, and 4hours, respectively. In the case of chapatti, it was found to be 89.84, 83.70, 81.36, and 76.92%, respectively. From the point of the interval of 1 hour each from the instant, it was prepared. Moreover, the solid-bound activity percentage with idli meal was 83.38, 81.07, 77.33, and 75.46% in similar intervals. This meal showed better results because of the good uniformity of ^99m^ Tc-SC inside solid particles of meal for which the meal preparation method has to credit which allowed the molecules to get proper spaces.

### Medium of Testing

Another factor responsible for radiolabeling efficiency is the medium in which the radiolabeling stability test is performed. It is also suggested in the experiment done by Knight 2 that stability testing in a gastric fluid with HCl only, without pepsin, may be incorrect such that GES meal testing in just acid might not be as stable as in simulated gastric fluid with pepsin.

So, it is to be kept in mind that HCl is not the proper substitute for gastric fluids. 12 A few studies' oatmeal showed good radiolabeling efficiency. However, in a study done by Laura A. Drubach et al, 13 it was observed that the radiolabeling efficiency of oatmeal in gastric fluids was not suitable where the percentage of activity bound to solid phase was 62.1±1.7 and 77.2±6.8 at 1 and 4hours, respectively. Similarly, in our results, we found relatively poor radiolabeling efficiency in the case of oatmeal that is found to be 78.75, 75.54, 70.22, and 67.81% at the time interval of 1, 2, 3, and 4hours, respectively, performed under the simulated gastric fluids that are HCl and pepsin.

### Type of Radionuclide Used

The radionuclide is chosen to prepare the radiolabeled meal that also alters the radiolabeling efficiency of the meal. In a study performed to find out the effect of radionuclide used in meal preparation, it was concluded that the LE of the particulate agents ( ^99m^ Tc-SC, ^99m^ Tc tin colloid, ^99m^ Tc Nano colloid, and ^99m^ Tc MAA) was between 90 and 100% and LE of ^99m^ Tc pertechnetate and ^99m^ Tc DTPA was between 60 and 80% at 90minutes. 14

In an experiment done by Tseng et al using ^99m^ Tc DTPA as a radionuclide, the traditional values of oatmeal-based GES were established and observed good correlation with cardinal gastroparesis symptoms within the Chinese population. ^14^

In our experiment, we used the ^99m^ Tc-SC as radionuclide in meal preparation as it is approved by the U.S. Food and Drug Administration for oral administration. In contrast, most other radiopharmaceuticals, including 111In radiopharmaceuticals, are not. 2

Considering the factors including uniformity of the radiotracer, state of the meal, method of preparation, which are responsible for the efficient binding of radionuclide to the food particles or the meal and clearly observing the results after application of required statistical tests on the data we got after experimenting with the defined methodology earlier, it was highlighted that egg white meal had shown us brilliant binding that was expected and along with that chapatti labeled with ^99m^ Tc-SC has proved that it can be worthy of being a better substitute to the egg white as it showed the radiolabeling binding efficiency of 92.01, 89.39, 86.73, and 84.47% at 1, 2, 3, and 4hours, respectively. The result of the idli meal in our experiment indicates that it can be a substitute to the standard egg white but lags behind the chapatti in terms of the effective binding with the radionuclide. Bread and butter showed relatively poor radiolabeling because there is a lack of uniformity in the meal. Oatmeal has gained popularity in the race of being a substitute to the standard egg white because of the easy preparation. It has again shown the comparatively inefficient binding compared with the other meals because of the nonuniformity of ^99m^ Tc-SC the components of the meal and being a semisolid meal also justifies the less binding efficiency in comparison to other meals that are all found to be in solid state.

The medium of testing and the type of radionuclide used in our experiment, which were simulated gastric fluid (pepsin and HCl) and ^99m^ Tc-SC, respectively, were the same for the all-sample meals, and that is the reason that the binding efficiency of the radionuclide we got for each meal can be easily compared.

In addition to that, after applying the correlation test on the experimental data, we learned that there was a strong correlation between all the radiolabeled meals prepared for our quantitative analysis.

The radiolabeled chapatti has emerged as an alternative solution for our dependency on the egg whites for conducting the GES. Our experiment has also opened up a new horizon to easily use a meal with rich nutritional value for GES. However, at the same time, it has further raised a question that despite showing better results in vitro where simulated gastric fluids were used, would be able to match the conditions inside the human stomach or not and what the patient's digestive responses would be of the patient for radiolabeled chapatti.

Moreover, after knowing how the type of radionuclide can alter the binding efficiency, there is a requirement of a study related to efficient binding of different types of radionuclide with the chapatti and idli meal as well. We know that egg whites bind better with ^99m^ Tc-SC but testing the radiolabeling efficiency on the substitutes such as chapatti and idli meal with the radionuclide other than ^99m^ Tc-SC such as tin colloid, nanocolloid, MAA, as well as ^99m^ Tc DTPA can further give us new dimension as it may or may not predict the much more efficient binding.

Earlier studies done at various institutes and our study have provided suggestions over the different alternatives, which can be a roadmap for establishing a list of meals that can be used in GES. It can benefit the patients as they would undergo the GES after eating the radiolabeled meal as per their desire and get treatment later. However, further studies are required before clearly opting out meals that can bind to a radionuclide well and on this aspect, attention is needed toward the various factors we have discussed above, and new characteristics must be explored.

## Conclusion

After analyzing the results from experiments, we can easily conclude that the LE of chapatti meal and idli meal is very much near eggs white's omelet and could be used as alternative to egg whites in vegetarian patients or patients who have an allergy to eggs. The other meals of bread along with butter and instant oatmeal meals did not show the desired results and cannot be used as an alternative to egg white as their LE is too low compared with standard egg whites.
